# 490. Identifying and Evaluating the Drivers of COVID-19 Vaccine Hesitancy Among Individuals with HIV in New Jersey

**DOI:** 10.1093/ofid/ofad500.559

**Published:** 2023-11-27

**Authors:** Jessica Occhiogrosso, Leena Rijhwani, Divya Rijhwani, Aravind Rajagopalan, Theresa C Fox, Roseann Marone, Gail Burack, Sunanda Gaur, Tanaya Bhowmick

**Affiliations:** Rutgers Robert Wood Johnson Medical School, Piscataway, New Jersey; Rutgers Robert Wood Johnson Medical School, Piscataway, New Jersey; Campbell University School of Osteopathic Medicine, Norwood, New Jersey; Rutgers Robert Wood Johnson Medical School, Piscataway, New Jersey; Rutgers, Trenton, New Jersey; Rutgers University Robert Wood Johnson Medical School, New Brunswick, New Jersey; Rutgers-Robert Wood Johnson Medical School, New Brunswick, New Jersey; Rutgers Robert Wood Johnson Medical School, Piscataway, New Jersey; Rutgers Robert Wood Johnson Medical School, Piscataway, New Jersey

## Abstract

**Background:**

Vaccine hesitancy, defined as the “delay in the acceptance or refusal of vaccines despite availability,” has been a significant challenge to vaccine uptake during the COVID-19 pandemic. Few studies have examined COVID-19 vaccine hesitancy among Americans living with HIV, a population with increased vulnerability to severe infection.

**Methods:**

We conducted a cross-sectional phone survey of 69 adults diagnosed with and receiving longitudinal care for HIV across 3 Ryan White HIV/AIDS Program-funded medical facilities in New Jersey. Surveys were administered from July 2022 to February 2023. Questions targeted COVID-19 vaccination status and concerns, factors that influenced vaccination decisions, and HIV stigma. Participant demographics and medical history were retrieved from the Ryan White Program’s CAREWare database. Data was summarized with descriptive statistics and bivariate correlations, and assessed for mean differences between “low” and “high” average vaccine hesitancy.

**Results:**

Among 69 participants, 96% received ≥1 COVID-19 vaccine and 81% received ≥1 booster (Table 1). 87% were motivated to get vaccinated because it was “necessary due to their health status,” and 70% cited the healthcare team as a “very significant” influence on opinions regarding COVID-19 vaccination. 37 of 69 participants reported higher average vaccine hesitancy (avg. rating > 2.5 out of 5) and thought the healthcare system was less likely to protect their privacy (p = 0.029). No significant difference was noted in HIV stigma, CD4 count, or years on ART between more vs. less hesitant groups. Additionally, participants who were strongly inclined to follow vaccine recommendations from their HIV care provider were more likely to believe that vaccination is important for disease prevention and protection (p < 0.001 for both), and more likely to have received a booster (p < 0.001).

**Figure:**
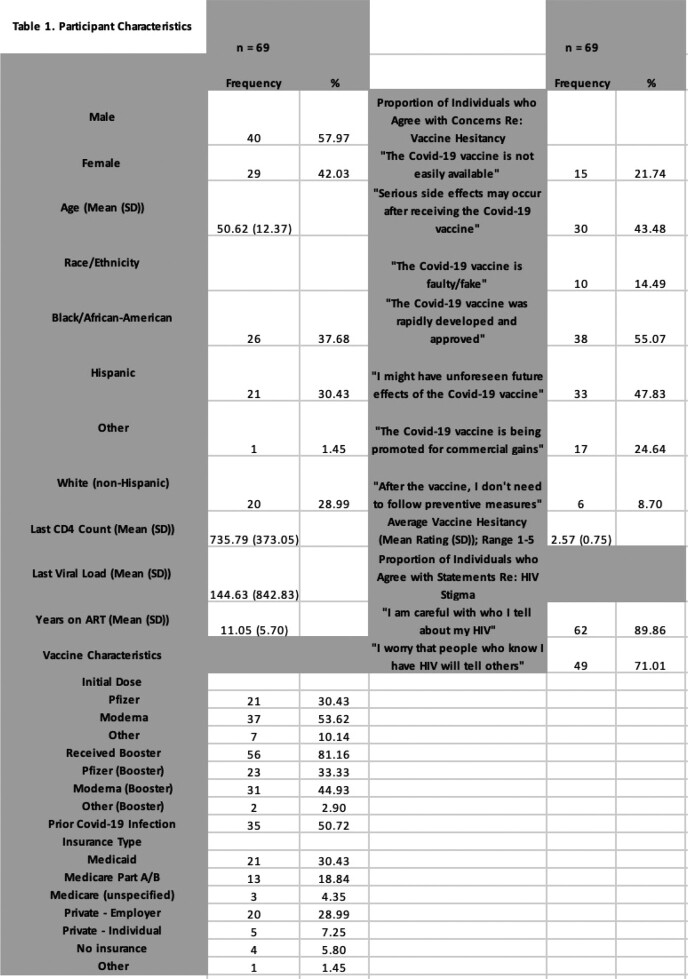

**Figure:**
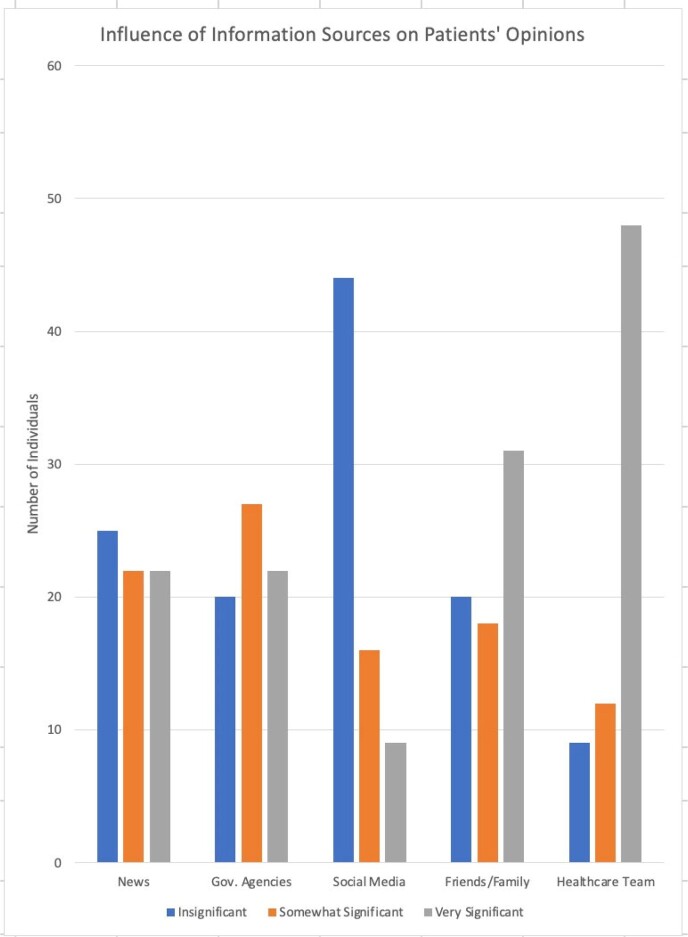

**Figure:**
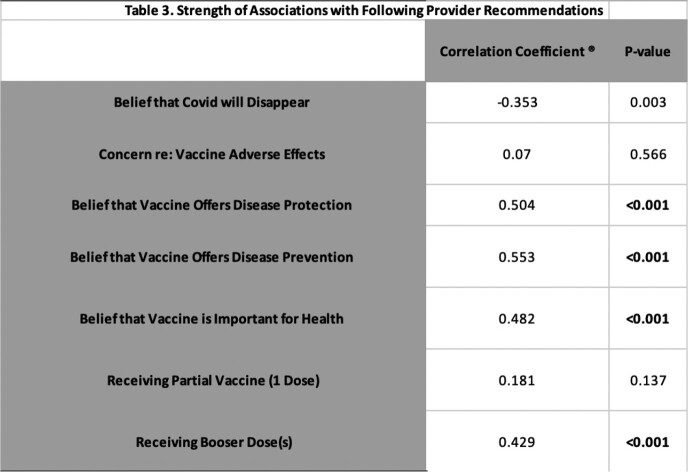

Table 3: strength of associations between those who reported to "generally follow" what their doctor or health care provider recommends about vaccines for their health and specific beliefs about vaccination.

**Conclusion:**

While most participants reported some degree of vaccine hesitancy, their level of hesitancy was not linked to disease-specific variables such as CD4 count, viral load, years on ART, or HIV stigma. However, most participants reported following vaccine recommendations from their HIV care provider, underscoring healthcare professionals’ critical role in vaccine education among people living with HIV.

**Disclosures:**

**Tanaya Bhowmick, MD**, Shinogi: Advisor/Consultant

